# Low frequency of the wild-type freezing-tolerance *LsCBF7* allele among lettuce population suggests a negative selection during domestication and breeding

**DOI:** 10.1007/s00122-024-04643-8

**Published:** 2024-05-18

**Authors:** Sunchung Park, Ainong Shi, Beiquan Mou

**Affiliations:** 1 U.S. Department of Agriculture, Agricultural Research Service, Beltsville, MD 20705 USA; 2 Horticulture Dept, University of Arkansas, Fayetteville, AR 72701 USA; 3 U.S. Department of Agriculture, Agricultural Research Service, Salinas, CA 93905 USA

## Abstract

**Key message:**

Sustainable winter production in lettuce requires freezing tolerant varieties. This study identified a wild-type allele of *LsCBF7* that could contribute to freezing tolerance improvement in lettuce.

**Abstract:**

Lettuce is one of the most consumed vegetables globally. While ideally grown in 13–21 °C, its cultivation extends into winter in milder climates. However, occasional freezing temperatures can significantly reduce yields. Therefore, the development of freezing-tolerant lettuce varieties has become a long-term goal of lettuce breeding programs. Despite its significance, our understanding of freezing tolerance in lettuce remains limited. Plants have evolved a coping mechanism against freezing, known as cold acclimation, whereby they can increase freezing tolerance when pre-exposed to low nonfreezing temperatures. The CBF pathway is well-known for its central role in cold acclimation. Previously, we identified 14 *CBF* genes in lettuce and discovered that one of them, *LsCBF7*, had a loss-of-function mutation. In this study, we uncovered that accessions from colder regions carried the wild-type allele of *LsCBF7* and this allele likely contributed to increased freezing tolerance, with 14% of the lettuce population carrying this allele. Interestingly, in wild lettuce (*L. serriola*) that is considered a progenitor of cultivated lettuce, this wild-type allele was much more common, with a frequency of 90%. This finding suggests that this wild-type allele may have undergone negative selection during the domestication or breeding of lettuce. Our data strongly indicate that this allele could be linked to early bolting, an undesirable trait in lettuce, which may have driven the negative selection. While this wild-type allele shows promise for improving freezing tolerance in lettuce, it is crucial to decouple it from the early bolting trait to fully harness its potential in lettuce breeding.

**Supplementary Information:**

The online version contains supplementary material available at 10.1007/s00122-024-04643-8.

## Introduction

Freezing temperature is a major environmental factor that restricts the geographical distribution of plants, leading to growth inhibition and yield loss in crops (Sakai and Larcher 1987; Yadav et al. 2020). To counter this freezing stress, plants have evolved diverse coping mechanisms. One such mechanism involves the enhancement of freezing tolerance when plants are exposed to nonfreezing low temperatures, a process known as cold acclimation (Thomashow 1999). While the precise mechanisms that underlie the enhancement of freezing tolerance through cold acclimation are not fully understood, many studies have suggested that C-repeat binding factors (CBFs) play a critical role in this process (Thomashow 2010; Park et al. 2015, 2018). In *Arabidopsis thaliana* (hereafter referred to as Arabidopsis), where the CBF pathway has been extensively studied, this pathway involves the action of three *CBF* genes encoding closely related AP2/ERF family transcription factors (Park et al. 2023). These three *CBF* genes are rapidly induced within minutes in response to low temperatures (Stockinger et al. 1997). They subsequently activate over 100 downstream target genes associated with metabolic, biochemical, and physiological processes that collectively enhance freezing tolerance (Gilmour et al. 2004; Cook et al. 2004; Kaplan et al. 2007; Li et al. 2020).

One important process during cold acclimation is the accumulation of raffinose family oligosaccharides (Taji et al. 2002; Pennycooke et al. 2003). This raffinose family plays a critical role in freezing tolerance by providing osmotic protection to plants, safeguarding chloroplasts, and stabilizing photosystem II against freezing damage (Knaupp et al. 2011). Galactinol synthase (GOLS), the enzyme catalyzing the initial and rate-limiting step in raffinose biosynthesis, plays a regulatory role in partitioning carbon between sucrose and raffinose family oligosaccharides (Saravitz et al. 1987). This gene is well-documented for its crucial role in freezing tolerance in various plant species including alfalfa (Cunningham et al. 2003), cucumber (Dai et al. 2022), *Ammopiptanthus* (Liu et al. 2020), *Medicago falcata* (Zhuo et al. 2013), *Petunia* (Zhang et al. 2022), and Arabidopsis (Taji et al. 2002).

In the Arabidopsis genome, there are at least seven *GOLS*-like genes, but only three of them, *AtGOLS1*, *AtGOLS2*, and *AtGOLS3*, respond to abiotic stresses (Taji et al. 2002). Among these, *AtGOLS1* and *AtGOLS2* were induced in response to water deficit, salinity, and heat stress by heat shock transcription factors (Panikulangara et al. 2004; Schramm et al. 2008; Nishizawa-Yokoi et al. 2009), while *AtGOL3* transcripts highly accumulated exclusively in response to cold stress. This cold-induction appears to be specifically regulated by the action of CBF transcription factors (Taji et al. 2002; Park et al. 2015). CBF-overexpressing plants induced only *AtGOLS3* among the seven family genes at warm temperatures, and the induction of *AtGOLS3* by low temperatures was significantly impaired in CBF loss-of-function mutants (Zhao et al. 2016; Park et al. 2018). Moreover, of the 14 first-wave transcription factors (CBF1-3, MYB73, CRF2, RAV1, CRF3, ERF5, DEAR1, MYB44, CZF1, ZAT10, ZF and HSFC1) that are rapidly induced during cold acclimation, none was capable of inducing *AtGOLS3* when overexpressed (Park et al. 2015). These findings indicate that the cold-induction of *AtGOLS3* relies solely on the CBF regulatory module in Arabidopsis, with *AtGOLS3* as a primary target of the CBF transcription factors.

The conservation of the CBF pathway is widespread among higher plants including lettuce, with many studies identifying CBF orthologous genes and highlighting their crucial roles in freezing tolerance (Owens et al. 2002; Skinner et al. 2005; Badawi et al. 2007; Carvallo et al. 2011; Hadi et al. 2011). The CBF genes have been identified as a quantitative trait locus (QTL) associated with freezing tolerance in crop species, making it a promising candidate for improving freezing tolerance in crops (Francia et al. 2007; Dumont et al. 2009; Li et al. 2013; Tayeh et al. 2013; Sieber et al. 2016; Adhikari et al. 2021). In fact, in natural populations, varying degrees of freezing tolerance were attributed to genetic variations in the CBF pathway. For example, Arabidopsis accessions originating from cooler northern latitudes generally displayed greater freezing tolerance compared to those from warmer southern latitudes (McKhann et al. 2008; Zhen and Ungerer 2008; Ågren and Schemske 2012). This clinal variation was mapped to the CBF locus in Arabidopsis, underscoring the pivotal role of CBF in adaptation to local climates (Ågren et al. 2013). Interestingly, the effect of CBF in local adaptation is reciprocal. Gain-of-function CBF variants are favored in colder regions, while they confer a disadvantage in warmer regions (Lee et al. 2024). Likewise, loss-of-function CBF alleles provide better fitness in warmer regions (Ågren and Schemske 2012; Ågren et al. 2013). Consistently, multiple studies in Arabidopsis found that accessions adapted to warmer regions carried nonfunctional CBF alleles, potentially contributing to adaptive evolution in the population (Kang et al. 2013; Gehan et al. 2015; Monroe et al. 2016). Although the precise mechanism underlying the diminished fitness associated with increased CBF activity in warmer climates remains elusive, it is postulated that erroneous activation of CBF, potentially triggered by fluctuating temperatures with a rare risk of freezing, could divert valuable resources away from growth-related processes in these warmer environments (Zhen et al. 2011; Oakley et al. 2014).

Lettuce (*Lactuca sativa*) is a leafy vegetable that offers numerous health benefits, including dietary fiber, minerals, and vitamins (Mou 2008). For year-round production, lettuce continues to be cultivated through the colder months in regions with mild winter temperatures, such as Imperial Valley, California, and Yuma, Arizona, which are major winter production areas in the USA (Geisseler and Horwath 2014). However, brief exposure to frost can cause lettuce leaves to blister and peel, making them vulnerable to decay and plant pathogens. This can significantly reduce lettuce yield and quality (Luna 2013; Boling 2022). Therefore, enhancing freezing tolerance has become a crucial, long-term objective in lettuce breeding for winter production.

Despite its importance, our understanding of freezing tolerance in lettuce is limited. A recent study by Park et al (2020) unveiled 14 CBF family genes in lettuce through a genome-wide search based on the reference genome constructed from the ‘Salinas’ cultivar (Reyes-Chin-Wo et al. 2017). Intriguingly, among these genes, one lettuce CBF gene, *LsCBF7*, was identified to have a loss-of-function mutation. This finding suggested that the mutant allele could potentially underlie freezing tolerance variation among the lettuce population. In this study, we investigated the allelic variation of the *LsCBF7* gene and its contribution to freezing tolerance in lettuce and explored the frequency distribution of different *LsCBF7* gene alleles among cultivated lettuce and wild lettuce populations. Furthermore, we addressed the potential of the wild-type allele as a promising candidate for enhancing freezing tolerance in lettuce. Our study sheds light on the intricate interplay between genetic variation and genetic trade-off, offering valuable insights for future breeding and improvement efforts in lettuce.

## Materials and methods

### Plant material and growth conditions

The lettuce genotypes used in this study consisted of 578 accessions maintained at the USDA-ARS, in Salinas, CA, USA, including crisphead, butterhead, leaf, romaine, primitive, and wild lettuce (Table S3).The lettuce plants were grown in pots with potting mix soil in a controlled growth chamber, where conditions were maintained at 20 °C with a 16 h photoperiod, except for the bolting time measurements. For bolting time measurements, plants were grown in a growth chamber with a day temperature of 33 °C and a night temperature of 19 °C. This temperature regime closely resembled the conditions used in the bolting experiment conducted by Park et al (2021). The plants were periodically rotated using a complete randomized block design. The light intensity was maintained in the range of 350 to 400 μmol m^−2^ s^−1^. For cold acclimation, plants were exposed to 4 °C for 24 h or 7 days with a light intensity of 100 μmol m^−2^ s^−1^.

### Identification of *GOLS* genes in lettuce through phylogenetic analysis

To identify *GOLS* genes and determine their orthology, a comprehensive approach involving protein databases from 15 plant species including lettuce was undertaken. Among these, nine species were from the Asterid and six were from the Rosid clade (Table S1). The protein databases except for lettuce were obtained from the NCBI database. The lettuce protein database (genome v8: id37106) was sourced from https://genomevolution.org/coge. Initially, the protein sequences of Arabidopsis *GOLS* family genes (i.e., *AtGOLS1-7*) were used as queries and the protein databases were searched using the BLASTP method with an e-value threshold of < 1e−20. This search resulted in the selection of 100 genes, which were subsequently subjected to phylogenetic analysis (Table S2).

To determine orthology among the selected genes, their protein sequences were aligned using ClustalW2 with default parameters, and further refinement of the alignment was performed, if necessary, using BioEdit (Hall 1999). A phylogenetic tree was generated based on the alignment using the neighbor-joining (NJ) method implemented in MEGA 11 (Tamura et al. 2021) with the parameters of Jones-Taylor-Thornton model, uniform rates among sites, and pairwise deletion of gaps. The resulting tree was visualized using FigTree (version 1.4.4) (http://tree.bio.ed.ac.uk/software/figtree).

### Freezing tolerance assay

To assess the freezing tolerance of the plants, electrolyte leakage assays were performed as described by Park et al (2015). The experiments were conducted using 28 day old plants. For cold acclimation, plants were subjected to 4 °C for 7 days under a 16 h photoperiod. Leaf disks were obtained by using a 1.5 mm diameter punch from the 4th or 5th leaf of two different plants and were placed in glass tubes. Each experimental condition was replicated three times. The tubes were then placed in a freezing chamber (Percival model LT-41VL, https://www.percival-scientific.com). To initiate the freezing process, the tubes were lowered to a temperature of − 2 °C in the dark. After an hour of equilibrium at − 2 °C, the leaf discs were frozen by adding ice-chips into the tubes. Following an additional 30 min, the tubes for the − 2 °C test were removed from the freezing chamber and placed on ice, and the temperature in the chamber was gradually lowered by 1 °C every 30 min until reaching − 12 °C. Once the specified temperatures were achieved, the tubes for each test temperature were taken out and thawed overnight on ice at 4 °C in the dark. To measure electrolytes leaked from leaf disks, 10 ml of deionized water was added into the tubes, which were then gently agitated for 4 h. After measuring the conductivity of the solutions, the solutions were transferred to new tubes, and the leaf discs were frozen at − 70 °C overnight. The next day, the preserved solutions were poured back to the frozen leaf disks and shaken for 4 h. The conductivity of the solutions was measured once again, and the ratio between the initial and subsequent conductivities was calculated.

### *LsCBF7* allele genotyping

SNP genotyping for the *LsCBF7* alleles was performed for 578 accessions (Table S3), with 441 of these accessions previously genotyped across the genome using the genotyping-by-sequencing (GBS) method (Park et al. 2021). The SNP genotype was conducted using the rhAmp SNP Genotyping System (Integrated DNA Technologies, https://www.idtdna.com). Primers were designed using the rhAmp-genotyping design tool (Table S4). Each genotyping assay consisted of 5 µL of rhAmp Genotyping Master Mix, 0.25 µL of rhAmp Reporter Mix, 0.5 µL of rhAmp SNP Assay, 2.25 µL of water, and 2 µL of template genomic DNA (10 ng). PCR reactions were conducted on a LightCycler 480 (Roche) following a cycling condition: enzyme activation for 10 min at 95 °C, followed by 40 cycles of amplification (95 °C for 10 s, 60 °C for 30 s, 68 °C for 20 s). The fluorescent signals were analyzed using Light Cycler 480 software according to the manufacturer’s instructions. The *LsCBF7* allele regions from at least 5 different accessions for each allele were sequenced to confirm the SNP.

### Quantitative real-time PCR

Total RNA was extracted from leaf tissues of 18-day-old lettuce seedlings using the RNeasy Plant Mini kits (Qiagen, http://www.qiagen.com/). cDNA synthesis was carried out with 200 ng of total RNA and random primers using the Reverse Transcription System (Promega, https://www.promega.com). Quantitative real-time PCR (qRT-PCR) was conducted using the fast SYBR Green master mix (Life Technologies, http://www.lifetechnologies.com). Two housekeeping genes, eukaryotic translation initiation factor 2A (*EIF2a; Ls6g95581*) and isopentenyl diphosphate isomerase 2 (*IPP2; Ls2g17540*), were used as reference genes following the method described by Park et al (2020). The primer sequences used for qRT‐PCR are listed in Table S4. Relative expression values were calculated using the ddCt method, where the average of the two reference genes served as a baseline, and normalized values were expressed as fold-changes relative to the control treatment.

### RNA-seq analysis

Leaf tissues were collected from cultivar ‘Salinas’ and ‘PI 284702’ plants exposed to 4 °C for 0 h, 24 h and 7 days. Total RNA was isolated using Qiagen Plant RNeasy kit (https://www.qiagen.com) and submitted to Novogene Corporation (https://en.novogene.com/) for RNA-seq. Sequencing was performed in a 150 bp paired-end format using the Illumina HiSeq platform (http://www.illumina.com). The RNA-seq reads were aligned to the *Lactuca sativa* reference genome (version 8) using STAR v2.5.2 (Dobin et al. 2013). The resulting alignments (BAM files) were processed to count gene-level reads using featureCounts (Liao et al. 2014). Differential expression analysis was conducted using the edgeR package in the R environment (Robinson et al. 2010). To mitigate the influence of lowly expressed genes, only those with a minimum of 0.5 reads per million (CPM) in at least two biological replicates were included in the analysis.

Our primary interest was in the differential gene expression resulting from the interaction between *LsCBF7* alleles and the cold-acclimation treatment. To investigate the interaction between genotype and cold-acclimation treatment on gene expression, we employed a generalized linear model in edgeR. Differences were expressed as log_2_-fold changes (FC), where the subscript ‘cold’ represents either 24 h or 7 days of cold treatment, as follows:

$${\text{FC}}_{{{\text{cold}}{\text{.PI284702}}}} - {\text{FC}}_{{{\text{cold}}{\text{.Salinas}}}} = \log_{2} \left( {\frac{{{\text{PI }}\,284702_{{{\text{cold}}}} }}{{{\text{PI}}\,{ }284702_{{{\text{warm}}}} }}} \right) - \log_{2} \left( {\frac{{{\text{Salinas}}_{{{\text{cold}}}} }}{{{\text{Salinas}}_{{{\text{warm}}}} }}} \right)$$

Genes with a two-fold change (log_2_ = 1) or greater, and a false discovery rate of 0.01, were considered differentially cold-responsive between Salinas and PI 284702. To identify genes analysespotentially regulated by *LsCBF7* in response to low temperatures, we further refined this gene set to include only those induced in PI 284702 during cold acclimation. Hierarchical clustering analyzes were performed using the hcluster method from the R package amap (R core team 2023). The resulting clusters were visualized with Treeview (http://rana.lbl.gov/EisenSoftware.htm). The RNA-seq data for PI 284702 have been deposited in the Gene Expression Omnibus under accession number GSE241604, and the RNA-seq data for Salinas was obtained from GSE134012.

### Bolting time measurement

To assess bolting time, a randomized complete-block design was employed, with at least five replicates for each individual accession. Bolting time was quantified as the number of days from the initial sowing to the point when the main stem reached a length of 5 cm.

### Genetic diversity between *LsCBF7* allelic groups and *L. serriola accessions*

The GBS-based SNP data for the 441 lettuce accessions was obtained from the European Variation Archive (Project Number: PRJEB40369) (Park et al. 2021). To investigate the genetic divergence between *L. sativa* accessions with the *LsCBF7* wild-type allele or the mutant allele, and *L. serriola* accessions, pairwise fixation index (F_ST_) values between the groups were calculated using the method of Weir and Hill (2002) implemented in the R package BEDASSLE (Bradburd 2013). F_ST_ serves as a metric for assessing the variation in allele frequencies between populations, ranging between 0 and 1. Genetic diversity within each population was measured using two methods: (1) the average expected heterozygosity for all loci in a population (Nei 1973) and (2) the frequency of shared alleles among all individuals in a population (Chakraborty and Jin 1993).

## Results

### Genetic variation in *LsCBF7* gene in lettuce

**Fig. 1:**
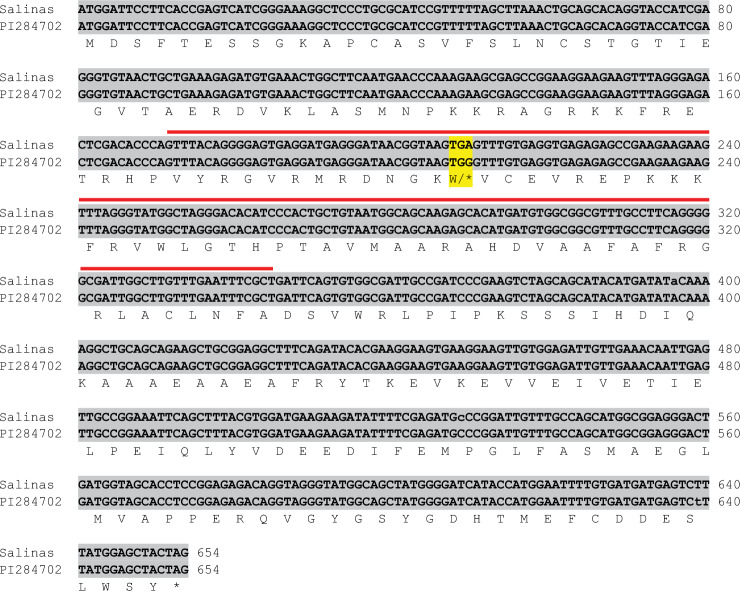
Alignment of nucleotide and amino acid sequences for the *LsCBF7* coding regions in the ‘Salinas’ and PI 284702 accessions. The predicted amino acid sequence is represented in single letter code, with the stop codon represented by an asterisk. Differences between the two accessions are highlighted in yellow; the A-to-G change at the 210th nucleotide results in a stop codon (*) changing to tryptophan (W). Red lines indicate the AP2 DNA-binding domain (color figure online)

In our earlier research, we identified a family of fourteen *CBF* genes in lettuce through a comparative phylogenetic analysis (Park et al. 2020). Among these genes, *LsCBF7* was found to have a premature stop within the AP2 DNA binding domain, resulting in a truncated and non-functional protein (Fig. 1). The expression analysis indicated that this mutant allele of *LsCBF7* was significantly induced in response to low temperature (4 °C), implying a role in cold stress responses in lettuce (Park et al. 2020). Natural variation in CBF genes has been reported in multiple plants, often correlated with variation in freezing tolerance (Francia et al. 2007; Dumont et al. 2009; Knox et al. 2010; Tayeh et al. 2013; Gehan et al. 2015; Sieber et al. 2016). These observations prompted us to investigate whether this mutant allele is associated with variation in the freezing tolerance of lettuce. Our hypothesis was that lettuce accessions adapted to colder regions might have wild-type alleles of *LsCBF7*, and the wild-type alleles could confer enhanced freezing tolerance to the lettuce accessions. To address this hypothesis, we sequenced the genomic DNA of the *LsCBF7* coding region for multiple accessions originating from colder climate, such as Sweden and Russia. Interestingly, our analysis identified a wild-type allele of *LsCBF7* within one of the accessions from Sweden, namely PI 284702 (Fig. 1). A specific single base difference (A-to-G) at position 210 bp within the coding region of *LsCBF7* resulted in a codon encoding tryptophan (W). This alteration led to the synthesis of a functional *LsCBF7* protein.

**Fig. 2:**
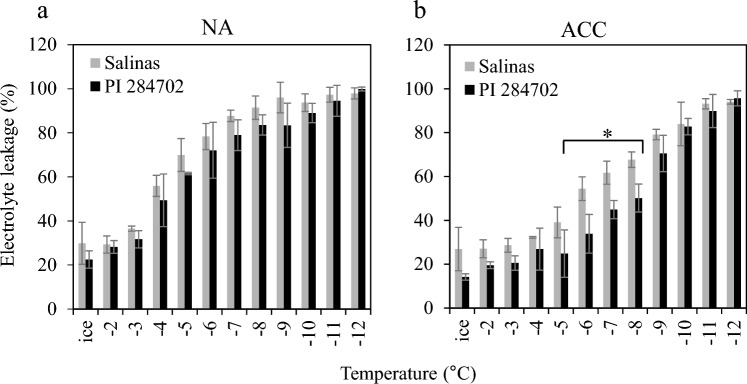
The PI 284702 accession exhibits greater freezing tolerance compared to the Salinas accession. Electrolyte leakage assays were conducted on non-acclimated (NA) plants grown at 20 °C and on cold-acclimated (ACC) plants (4 °C for 7 days). Error bars indicate standard error of three biological replicates. An asterisk indicates statistically significant differences between two accessions (*P* < 0.05, student *t*-test)

To assess the potential impact of the putatively functional wild-type *LsCBF7* allele on freezing tolerance, we conducted a freezing tolerance assay for PI 284702 (carrying the wild-type allele of *LsCBF7*) and ‘Salinas’ plants (carrying the mutant *LsCBF7* allele). The freezing tolerance assay was performed with and without cold acclimation at 4 °C for 7 days to assess the influence of cold acclimation on freezing tolerance in lettuce. Our electrolyte leakage assays showed minimal disparities between non-acclimated PI 284702 and ‘Salinas’ plants, both exhibiting an EL_50_ of around –4 °C (the temperature at which freezing damage results in leakage of 50% of the total electrolytes) (Fig. 2a). However, after cold acclimation, the PI 284702 plants exhibited significantly greater freezing tolerance compared to the ‘Salinas’ plants. Specifically, the EL_50_ of cold-acclimated PI 284702 plants improved to − 8 °C, while ‘Salinas’ plants showed an EL50 of approximately − 6 °C (Fig. 2b). Therefore, the cold acclimation contributed to 4 °C enhancement in freezing tolerance (from − 4 to − 8 °C) for PI 284702 plants, whereas ‘Salinas’ plants exhibited 2 °C improvement (from − 4 to − 6 °C). The increase in freezing tolerance due to cold acclimation was reduced by about 50% in the ‘Salinas’ plants. These results suggested that the loss of CBF function might account for the 2 °C reduction in freezing tolerance, potentially underlying the variance in freezing tolerance between these two accessions.

### Cold transcriptome analysis reveals *LsGOLS1* gene as a primary target of *LsCBF7*

**Fig. 3:**
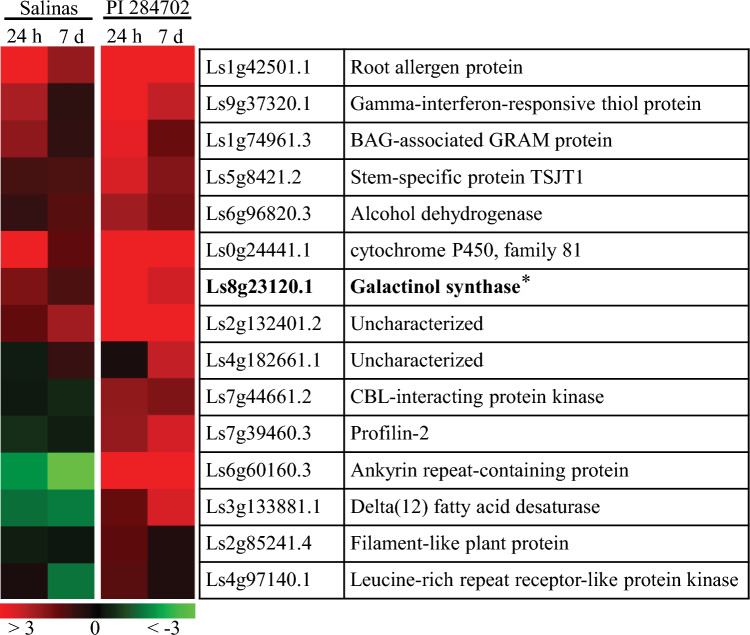
Heatmap for the differentially expressed genes during cold acclimation in Salinas and PI 284702. It shows the log_2_-fold change in transcript levels for the upregulated genes in PI 284702 plants relative to ‘Salinas’ plants after exposure to low temperature (4 °C) for 24 h or 7 days. Transcript levels in cold conditions were normalized by those in warm conditions (20 °C). The color scale represents log_2_-fold change

In response to low temperatures, CBF transcription factors play a pivotal role by upregulating an array of downstream target genes, which can orchestrate vital metabolic and biochemical processes that lead to the enhancement of freezing tolerance in plants (Gilmour et al. 2004; Cook et al. 2004; Kaplan et al. 2007). Therefore, the potential consequences of the loss-of-function mutation in *LsCBF7* on cold acclimation can be reflected in the expression levels of its downstream target genes. To investigate the impact of the *LsCBF7* mutation on the cold-induced expression of its targets, we performed RNA-seq experiments for PI 284702 and ‘Salinas’ plants subjected to low temperatures (4 °C) for 24 h and 7 days. The cold transcriptome analysis identified 15 genes that displayed significant differential expression between the two cultivars (Fig. 3; Table S5). Among these, one gene appeared to have substantial sequence similarity to a well-known CBF target—galactinol synthase, a gene widely recognized for its activation by CBF transcription factors in Arabidopsis (Park et al. 2015, 2018). In Arabidopsis, the *GOLS* genes belong to a multigene family with seven members of genes, with *AtGOLS3* being the only gene significantly activated by CBFs in response to cold stress (Taji et al. 2002; Gehan et al. 2015).

**Fig. 4:**
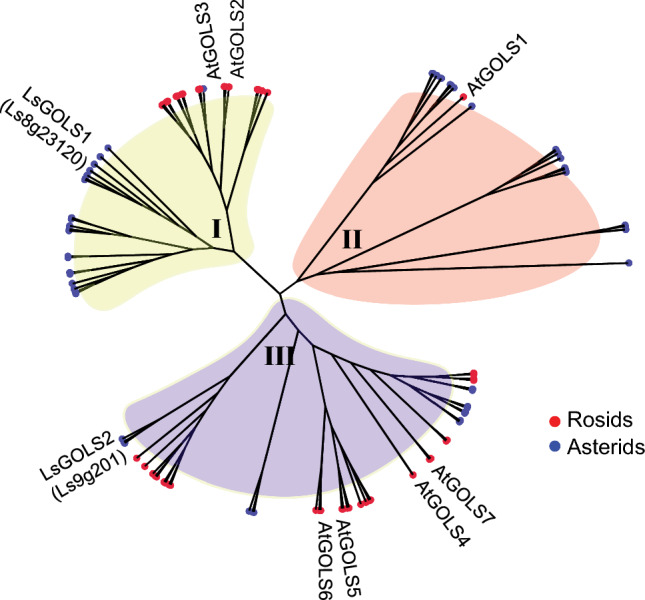
Phylogenetic analysis of 100 GOLS genes from 15 species, including lettuce and Arabidopsis. The tree was constructed based on the protein sequences using the neighbor-joining method. The nine species from the Asterid clade, including lettuce are marked with blue circles, and the six species from the Rosid clade, including Arabidopsis are marked with red circles. GOLS genes only from Lettuce and Arabidopsis are annotated in the tree (refer to Table S1 for genes from other species). Three ancestral groups are denoted I, II, and III

To examine whether a similar regulatory relationship exists in lettuce, we first conducted a comprehensive search for GOLS genes within the lettuce genome. Our analysis indicated the presence of two GOLS genes in lettuce. Subsequently, we determined the orthologous relationships between the GOLS genes in Arabidopsis and lettuce through a comparative phylogenetic analysis. Our phylogenetic analysis revealed three ancestral groups within the GOLS gene family in higher plants, designated as I, II, and III (Fig. 4). All three groups contained genes from both Asterid and Rosid clades of flowering plants, indicating that ancestral genes of these groups predate the separation of Asterid and Rosid clades. The two lettuce GOLS genes, named LsGOLS1 and LsGOLS2, were assigned to groups I and III, respectively, while at least one of the GOLS genes from Arabidopsis was assigned to each of the three groups: AtGOLS2 and AtGOLS3 belonged to group I, AtGOLS1 to group II, and ATGOLS4, ATGOLS5, ATGOLS6, and ATGOLS7 belonged to group III. Therefore, based on the phylogenetic tree, LsGOLS1 (Ls8g23120) was closely related to both AtGOLS2 and AtGOLS3 that are downstream target of AtCBFs. On the other hand, LsGOLS2 (Ls9g201) was orthologous to four other Arabidopsis genes: AtGOLS4–7 (Table S2).

**Fig. 5:**
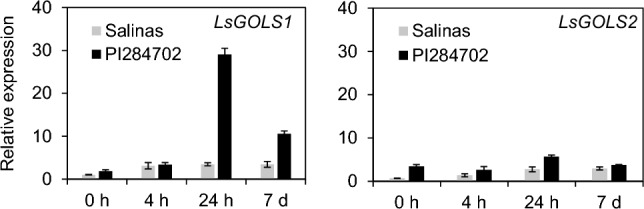
Expression of lettuce *GOLS* genes in response to low temperatures. Expression levels of *LsGOLS1* and *LsGOLS2* were determined by qRT-PCR in plants grown at 20 °C (0 h) and exposed to 4 °C for 4 h, 24 h and 7 days. Error bars represent standard error of three biological replicates

This orthologous relationship aligned with our RNA-seq results, where only *LsGOLS1* (*Ls8g23120*) was identified as cold-induced in PI 284702 (Fig. 3). Subsequent qRT-PCR corroborated this, confirming significant cold induction of *LsGOLS1* and minimal, if any, induction of *LsGOLS2* in response to low temperatures. The degree of cold induction for *LsGOLS1* was significantly higher in PI 284702 compared to ‘Salinas’, whereas *LsGOLS2* exhibited no significant difference in cold induction between the two cultivars (Fig. 5). This result strongly supported the idea that *LsGOLS1* is likely a direct target of *LsCBF7*, as in Arabidopsis, and the loss-of-function *LsCBF7* mutation dampened the cold-induced expression of *LsGOLS1* in ‘Salinas’ plants. Given the important role of GOLS genes in freezing tolerance, this finding supported the conclusion that the loss-of-function *LsCBF7* mutation led to reduced freezing tolerance in ‘Salinas’ plants.

### *LsCBF7* allele frequency varies among lettuce horticultural types and wild lettuce

**Fig. 6:**
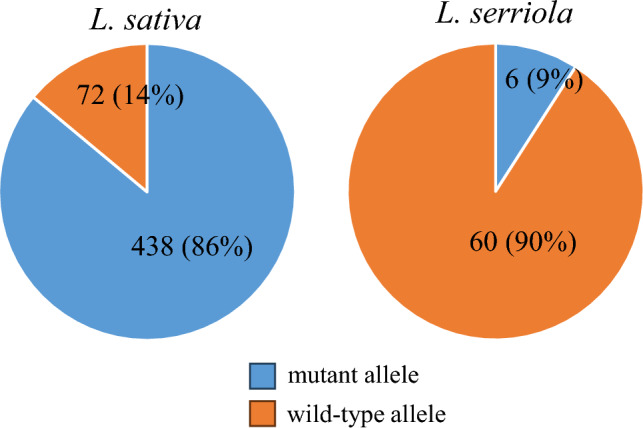
Distribution of different *LsCBF7* allele among cultivated lettuce (*L. sativa*) and wild lettuce (*L. serriola*). The numbers of accessions are shown on the pie chart, along with their respective percentages in parentheses

As the accession (PI 284702) with the wild-type *LsCBF7* allele exhibited greater freezing tolerance compared to the accession (Salinas) with the mutant allele, we sought to investigate further, with a larger set of accessions, whether genetic variation in the *LsCBF7* gene could contribute to difference in freezing tolerance. To this end, we genotyped *LsCBF7* allele polymorphisms for 578 accessions, including four main horticultural types—115 crisphead, 118 romaine, 116 leaf, 136 butterhead—as well as 67 wild lettuce *L. serriola* accessions (Table S3). Our data revealed that the mutant *LsCBF7* allele was predominant within the lettuce population, representing 86% (438) of the sampled *L. sativa* accessions, while only 14% (72) of the accessions carried the wild-type allele. Interestingly, the frequency of the wild-type allele varied among the four main horticultural types: 25% of leaf type accessions, 15% of romaine type, 10% of butterhead type, and 1% of crisphead type (Table 1). Furthermore, we observed a striking difference in the frequency of the *LsCBF7* alleles among *L. serriola* accessions, the species that were commonly believed to be the progenitor of domesticated lettuce, *L. sativa*. In *L. serriola*, 90% (60) of accessions had the wild-type *LsCBF7* allele, while only 9% (6) had the mutant allele, and one accession was heterozygous (Fig. 6). This significant discrepancy (binomial test, *P* value < 2.2e−16) suggested that the wild-type *LsCBF7* allele may have been negatively selected during lettuce domestication or breeding programs.

**Table 1:** Distribution of *LsCBF7* allele genotype among four horticulturals types of lettuce

Horticultural type	Allele genotype*			% (Wt/total) (%)
	Wt	Mt	Ht	
Leaf	29	87	0	25
Romaine	18	99	1	15
Butterhead	14	122	0	10
Crisphead	1	114	0	1

*Wt represents wild-type; Mt, mutant; Ht, heterozygous

### *LsCBF7* allelic variation likely contributes to freezing tolerance variation among lettuce population

**Fig. 7:**
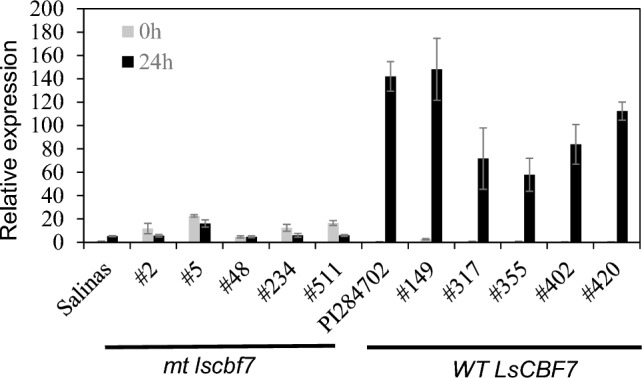
*LsGOLS1* expression in response to low temperatures in accessions carrying mutant or wild-type *LsCBF7* alleles. Five accessions were randomly selected for each mutant (*mt lscbf7*) and wild-type (*WT LsCBF7*) allele group to assess *LsGOLS1* expression. ‘Salinas’ and PI 284702 were included as controls. Accession identification numbers are shown on the *x*-axis. Gene expression was determined by qRT-PCR in plants grown at 20 °C (0 h) and exposed to 4 °C for 24 h. Error bars represent the standard error of three biological replicates

To investigate the association between genetic variation in the *LsCBF7* gene and variation in freezing tolerance among lettuce accessions, we initially examined the cold-induced expression of *LsGOLS* genes in the additional accessions with different *LsCBF7* genotypes. We randomly chose five accessions, each for the wild-type allele and the mutant allele of *LsCBF7*, and assessed the expression levels of *LsGOLS1* and *LsGOLS2* in plants exposed to a temperature of 4 °C for 24 h (Fig. 7). Consistent with our earlier findings from PI 284702 and ‘Salinas’ plants, the accessions with the wild-type *LsCBF7* allele exhibited significant induction of *LsGOLS1* expression following 24 h of cold treatment. In contrast, those carrying the mutant allele showed little or no induction of *LsGOLS1*. The expression levels of *LsGOLS2*, however, did not significantly differ between the two genotype groups (Fig. S1). These observations provided compelling evidence that *LsGOLS1* is a primary downstream target of the *LsCBF7* and its activation in response to low temperatures is dependent on *LsCBF7* function.

**Fig. 8:**
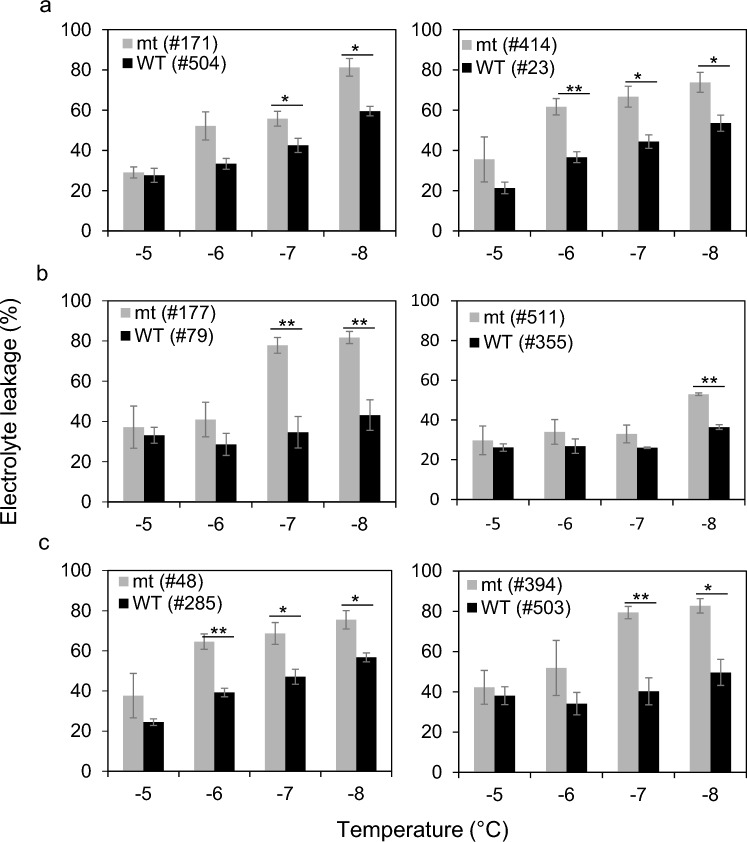
Loss-of-function mutation of *LsCBF7* impairs freezing tolerance in lettuce plants. Electrolyte leakage freeze tests were conducted on cold-acclimated plants exposed to 4 °C for 7 days. Accessions from the butterhead type (**a**), leaf type (**b**), and romaine type (**c**) carrying either mutant (mt) or wild-type (WT) *LsCBF7* allele were compared. Error bar indicates the standard error of three biological replicates. Significance was tested by Student’s *t*-test (**< 0.01, *< 0.05)

We further assessed the degree of freezing tolerance in these two genotype groups of accessions using electrolyte leakage assay. Different horticultural types have distinct growth characteristics, such as leaf texture and growth rate, which could potentially affect the assays. To minimize this possible bias, we selected accessions from the same horticultural types and tested them at a temperature range of − 5 to − 8 °C, which appeared to yield the most reliable results in electrolyte leakage assays at the earlier test with PI 284702 and ‘Salinas’ accessions (Fig. 2). In all six comparisons, the accessions carrying the wild-type *LsCBF7* allele showed greater freezing tolerance following cold acclimation compared to those with the mutant allele (Fig. 8). These results further substantiated the idea that the wild-type *LsCBF7* allele contributed to an increase in freezing tolerance in lettuce.

### The *LsCBF7* wild-type allele is associated with early bolting

Our genotyping analysis revealed a stark difference in the frequency of the wild-type *LsCBF7* allele between *L. sativa* and *L. serriola* accessions (Fig. 6). Given that lettuce (*L. sativa*) is widely believed to be domesticated from *L. serriola*, this discrepancy led us to hypothesize that the wild-type allele might have been subjected to negative selection during domestication process or breeding efforts. Notably, we observed a strong negative correlation between bolting time and the wild-type allele frequency for the four lettuce horticultural types, with *R*^2^ of 0.96 (Fig. S2). We compared the average bolting time of the four horticultural types obtained from Park et al. (2021), with their *LsCBF7* wild-type allele frequencies. The leaf type with the shortest average bolting time exhibited the highest frequency of the wild-type allele. The romaine type with the second-shortest bolting time showed the second-highest frequency, followed by butterhead and crisphead (Fig. S2). This finding suggested a potential association between the wild-type allele and early bolting trait.

Early bolting is generally considered an undesirable trait in lettuce cultivation, as it reduces the biomass of the leafy vegetable and leads to a bitter taste. Therefore, this association could be a driving force for the negative selection of the wild-type allele. To explore this idea, we referred to a phylogenetic tree previously constructed based on 186,000 SNP data with 441 accessions (Park et al. 2021), which were also genotyped for the *LsCBF7* alleles in this study (Table S3). Using the neighbor-joining tree as a guide, we selected eight pairs of genetically closely related accessions that carried different *LsCBF7* allele genotypes (Fig. S3). This approach was designed to minimize the influence of other genes on the bolting trait. We then grew these paired accessions under identical conditions and monitored their phenotypes. Our observations revealed that accessions carrying the wild-type *LsCBF7* allele exhibited earlier bolting compared to those with mutant alleles in seven out of eight pairs, while one pair showed similar bolting time between the different genotypes (Fig. S4). This finding strongly supported the notion that the wild-type *LsCBF7* allele is associated with the undesirable trait of early bolting, which could lead to the negative selection of the wild-type allele during domestication or breeding programs.

### Genetic relationship between the two *LsCBF7 *allelic groups of *L. sativa* and wild lettuce (*L. serriola*)

To gain insights into the genetic relationship between the two *LsCBF7* allelic groups of *L. sativa* and wild lettuce (*L. serriola*), we examined genetic similarity between these three groups and genetic diversity within the two allelic groups of *L. sativa*. The extent of genetic similarity was estimated using pairwise F_ST_ values, where lower F_ST_ indicates greater genetic similarity (Weir and Hill 2002). When the two allelic groups of *L. sativa* were compared to *L. serriola* accessions, the wild-type *LsCBF7* accessions showed a lower F_ST_ value (0.242) than the mutant accessions (0.263). When F_ST_ was estimated only for the chromosome 9 region where *LsCBF7* gene is located, difference between the two groups became more pronounced, with wild-type accessions displaying F_ST_ of 0.205 and mutant accessions having 0.286. These results indicated that accessions with the wild-type *LsCBF7* allele were genetically closer to *L. serriola* than those with the mutant allele, particularly in the context of the chromosome 9 region.

In addition, we estimated genetic diversity within the two allelic groups of *L. sativa*. The extent of genetic diversity was estimated as expected heterozygosity (H_exp_) (Nei 1973) and shared allele frequency (SAF) (Gao and Martin 2009), where higher H_exp_ and SAF indicates greater diversity within a population. The wild-type accessions exhibited higher H_exp_ (0.28) and SAF (0.54) values than the mutant accessions with H_exp_ of 0.26 and SAF of 0.50. When this estimation was limited to the chromosome 9 region, the genetic diversity for the wild-type accessions was even more pronounced. The wild-type accessions showed H_exp_ of 0.28 and SAF of 0.56, while the mutant accessions displayed H_exp_ of 0.23 and SAF of 0.44. These results indicated that accessions with the wild-type allele were genetically more diverse compared to accessions with the mutant allele.

## Discussion

The CBF pathway plays a prominent role in cold acclimation and has been identified as a freezing tolerance QTL in many plant species (Francia et al. 2007; Dumont et al. 2009; Li et al. 2013; Tayeh et al. 2013; Sieber et al. 2016; Adhikari et al. 2021). Previously, we discovered a loss-of-function mutation in one of the lettuce CBF family genes (Park et al. 2020). In this study, we further investigated the potential impact of this mutant allele on freezing tolerance. Our results demonstrated that cold acclimation in lettuce can increase freezing tolerance by 4 °C, and the mutant allele might cause the reduction of this increase by 50% compared to its wild-type allele. Additionally, we unveiled variations in the frequency of the wild-type allele among horticultural types. Notably, these variations displayed a strong negative correlation with the average bolting time of these lettuce types, with the higher frequency of the wild-type allele leading to earlier bolting. This observation suggested an association between the wild-type allele and early bolting trait. This notion was further substantiated by bolting time tests conducted on genetically similar pairs of accessions but with different *LsCBF7* alleles. Furthermore, we found a striking difference in the prevalence of the wild-type allele between cultivated lettuce (*L.sativa*) and wild lettuce (*L. serriola*). Only 14% of the *L.sativa* population carried the wild-type allele, while 90% of the *L. serriola* population carried the wild-type allele. Given that *L. sativa* is derived from *L. serriola*, our findings suggested that the wild-type allele underwent negative selection during the domestication of lettuce, despite its positive effect on freezing tolerance. We proposed that the negative selection of the wild-type allele might be driven by its association with early bolting. These results provide insights into how the genetic trade-off between freezing tolerance and early bolting, whether arising from linkage drag or pleiotropic effects, has played a role in the genetic make-up of lettuce during its domestication and breeding process.

Among the 14 CBF genes in lettuce, twelve were significantly induced in response to cold stress, suggesting their roles in freezing tolerance (Park et al. 2020). Nonetheless, the intriguing observation in this study is that a single CBF mutation in lettuce may significantly reduce the cold-induction of *LsGOLS1*. In Arabidopsis, the three CBFs are known to be functionally redundant, capable of inducing a similar set of target genes when overexpressed (Gilmour et al. 2004; Park et al. 2015). However, there is also evidence that they are not entirely redundant. Single or double mutations in the three Arabidopsis CBF genes still reduced freezing tolerance, although the effect was weaker compared to the triple CBF mutation (Zhao et al. 2016; Park et al. 2018), indicating that each of the three CBF transcription factors has its own non-redundant function. Consistently, CBF2 appears to have a more prominent influence on freezing tolerance than the other CBF genes in Arabidopsis. CBF2 overexpression activated the downstream target genes more strongly (Park et al. 2015)**,** and double mutations including *cbf2* (*cbf1/cbf2* and *cbf2/cbf3*) showed a greater reduction in freezing tolerance than other double mutations without *cbf2* (*cbf1/cbf3*) (Zhao et al. 2016). Therefore, these observations suggest that there may be some degree of non-redundancy among Arabidopsis CBF genes. In addition, when natural CBF mutations were surveyed across natural Arabidopsis populations, *cbf2* mutants were restricted to much warmer regions than other CBF mutants (Monroe et al. 2016), indicating that CBF2 mutation has a more significant impact on freezing tolerance. Taken together, these observations support the notion that *LsCBF7*, like CBF2 in Arabidopsis, may play a more prominent role among the lettuce CBF family, and its mutation alone can lead to the significant reduction in the cold-induced expression of *LsGOLS1*.

Our results revealed intriguing variations in the frequency of the *CBF* wild-type allele across different horticultural lettuce types. Particularly in crisphead type, only one accession carried the wild-type *LsCBF7* allele, while at least 10–25% of other horticultural types—butterhead, romaine, and leaf—possessed the wild-type allele. This distinction in the crisphead type may be attributed to a population bottleneck effect. Crisphead lettuce, the most recent development among the four types, originated from the French Batavia types in the twentieth century (de Vries 1997). Since then, most modern crisphead varieties have been developed in the United States from three cultivars (Calmar, Salinas, and Vanguard) as parental lines (Mikel 2007). Consistently, a study by Park et al. (2021) highlighted higher homogeneity within the crisphead type due to the extensive use of a small number of parents in a short period. Therefore, it is conceivable that the crisphead type may have originated from parents already possessing the mutant *LsCBF7* allele, contributing to this frequency pattern. In addition, positive selection for delayed bolting may also play a role in maintaining the near-absence of the wild-type allele in the crisphead type. Compact head formation is a distinguishing feature of crisphead lettuce, and early bolting disrupts this head formation, resulting in more severe yield loss compared to other types. Therefore, the crisphead type is under stronger selection pressure for delayed bolting. In fact, the crisphead type exhibited the most pronounced delay in bolting among the four types (Park et al. 2021). Given the association of the wild-type allele with early bolting, this selection pressure would prevent the introduction of the wild-type allele into the crisphead population.

While the wild-type allele is dominant among *L. serriola* population, the presence of the mutant allele in some wild accessions raised a question about its origin. There are two possible scenarios: (1) the mutation occurred in *L. serriola* and was inherited by cultivated lettuce (*L. sativa*), or (2) the mutation occurred in cultivated lettuce and transferred back to *L. serriola*, as the two species are cross-compatible. We propose that the latter scenario is more likely. In nature, *L. serriola* plants typically germinate in late fall and overwinter in a rosette stage (Prince et al. 1978). Therefore, the wild-type *LsCBF7* allele would provide a survival advantage during cold winter months, which subsequently allows for a high frequency of the wild-type allele within the population.

Considering the predominance of the wild-type allele in the *L. serriola* population, it is plausible that earlier domesticated lettuce would also carry the wild-type allele. Lettuce plants are typically cultivated during the warm growing season, avoiding the cold winter. Therefore, the beneficial effect of the wild-type allele on cultivated lettuce is not as pronounced as it is on wild lettuce, and the selection pressure on the wild-type allele would be relaxed. This relaxation may have allowed for the occurrence of the mutant allele, as observed in natural Arabidopsis populations adapted to warmer climates (Kang et al. 2013; Gehan et al. 2015; Monroe et al. 2016). Furthermore, the wild-type *CBF* allele might be less advantageous than the mutant allele in warmer regions that frequently experience temperatures low enough to induce *CBF* genes, but rarely experiencing freezing temperatures. False activation of CBF transcription factors could consume resources that could otherwise be utilized for growth, with the rare risk of freezing (Zhen et al. 2011; Oakley et al. 2014). Nevertheless, studies involving natural Arabidopsis populations suggest that the fitness benefit gained by the mutant allele may not be sufficient to account for its prevalence among cultivated lettuce in this study. For example, a survey for adaptive CBF mutations in warmer regions by Monroe et al. (2016) found an excess of nonsynonymous polymorphisms in *CBF* genes, but only 6% of the population carried nonfunctional mutations, with the functional *CBF* alleles still prevailing in the population. While previous studies (Ågren et al. 2013; Lee et al. 2024) found limited phenotypic effects of CBF loss-of-function mutations in warmer regions, primarily on fecundity, we observed a significant difference in bolting time between accessions carrying the wild-type and mutant alleles. This finding suggests that there could be a more influential factor contributing to the prevalence of the mutant allele within the lettuce population, such as delayed bolting.

Our bolting experiments (Figs. S2, S4) suggested that the mutant allele was associated with delayed bolting. Early bolting could be crucial for the survival and proliferation of wild lettuce, as it allows plants to produce seeds before the onset of unfavorable environmental conditions. On the other hand, delayed bolting in cultivated lettuce is a desirable trait with multiple benefits including increased yield and better quality, and this trait has been continuously selected in lettuce breeding programs (de Vries 1997; Hartman et al. 2013; Han et al. 2021). Therefore, during domestication or breeding, the association of the mutant allele with the delayed bolting trait accelerated the genetic fixation of the mutant allele among the modern lettuce population.

Our genetic diversity and similarity analyzes among the three genetic groups—*L. serriola*, accessions with the wild-type allele, and accessions with the mutant allele—also supported the idea that the wild-type allele was inherited from *L. serriola* and the mutant allele might be under positive selection in lettuce population. Our F_ST_ analysis indicated that accessions with the wild-type allele were genetically closer to *L. serriola* than those with the mutant allele (Table S6), making it more likely that the wild-type allele is passed down from *L. serriola* rather than the mutant allele. Moreover, the mutant accessions are genetically more homogeneous than the wild-type accessions, according to our H_exp_ and SAF analyzes (Table S6), suggesting that the mutant allele might be under strong selection, thereby resulting in reduced genetic diversity.

Although future empirical work will be needed to determine the nature of the association between the wild-type allele and early bolting—whether it is due to physical linkage or arises from a pleiotropic function of the wild-type allele—the utilization of this beneficial allele for improving freezing tolerance in lettuce would require decoupling the undesirable trait from the wild-type allele. Modern plant breeding techniques, such as CRISPR (clustered regularly interspaced short palindromic repeats), could also be utilized to modify the mutant allele and restore the cold tolerance trait in lettuce.

## Supplementary Information

Below is the link to the electronic supplementary material.

Supplementary file 1 (DOCX 327 kb)

Supplementary file 2 (XLSX 58 kb)

## Data Availability

The RNA-seq data are available in the Gene Expression Omnibus (www.ncbi.nlm.nih.gov/geo/) under accession number GSE241604. All relevant data are included in the manuscript and the Supporting Information files.
