# Correction to: Low frequency of the wild‑type freezing‑tolerance *LsCBF7* allele among lettuce population suggests a negative selection during domestication and breeding

**DOI:** 10.1007/s00122-024-04727-5

**Published:** 2024-09-23

**Authors:** Sunchung Park, Ainong Shi, Beiquan Mou

**Affiliations:** 1 U.S. Department of Agriculture, Agricultural Research Service, Beltsville, MD 20705 USA; 2 Horticulture Dept, University of Arkansas, Fayetteville, AR 72701 USA; 3 U.S. Department of Agriculture, Agricultural Research Service, Salinas, CA 93905 USA

**Correction to: Theoretical and Applied Genetics (2024) 137:135** 10.1007/s00122-024-04643-8

The article “Low frequency of the wild‑type freezing‑tolerance LsCBF7 allele among lettuce population suggests a negative selection during domestication and breeding”, written by Sunchung Park, Ainong Shi, and Beiquan Mou, was originally published electronically on the publisher’s internet portal on 18 May 2024 without open access. With the author(s)’ decision to opt for Open Choice the copyright of the article changed on 16 August 2024 to © The Author(s) 2024 and the article is forthwith distributed under a Creative Commons Attribution 4.0 International License, which permits use, sharing, adaptation, distribution and reproduction in any medium or format, as long as you give appropriate credit to the original author(s) and the source, provide a link to the Creative Commons licence, and indicate if changes were made. The images or other third party material in this article are included in the article’s Creative Commons licence, unless indicated otherwise in a credit line to the material. If material is not included in the article’s Creative Commons licence and your intended use is not permitted by statutory regulation or exceeds the permitted use, you will need to obtain permission directly from the copyright holder. To view a copy of this licence, visit http://creativecommons.org/licenses/by/4.0.

**Open Access** This article is licensed under a Creative Commons Attribution 4.0 International License, which permits use, sharing, adaptation, distribution and reproduction in any medium or format, as long as you give appropriate credit to the original author(s) and the source, provide a link to the Creative Commons licence, and indicate if changes were made. The images or other third party material in this article are included in the article’s Creative Commons licence, unless indicated otherwise in a credit line to the material. If material is not included in the article’s Creative Commons licence and your intended use is not permitted by statutory regulation or exceeds the permitted use, you will need to obtain permission directly from the copyright holder. To view a copy of this licence, visit http://creativecommons.org/licenses/by/4.0/.

