# Correction to: Low frequency of the wild‑type freezing‑tolerance *LsCBF7* allele among lettuce population suggests a negative selection during domestication and breeding

**DOI:** 10.1007/s00122-024-04783-x

**Published:** 2024-12-13

**Authors:** Sunchung Park, Ainong Shi, Beiquan Mou

**Affiliations:** 1 U.S. Department of Agriculture, Agricultural Research Service, Beltsville, MD 20705 USA; 2 Horticulture Dept, University of Arkansas, Fayetteville, AR 72701 USA; 3 U.S. Department of Agriculture, Agricultural Research Service, Salinas, CA 93905 USA

**Correction to: Theoretical and Applied Genetics (2024) 137:135** 10.1007/s00122-024-04643-8

The Fig. 6 has been incorrectly published in the original publication. The complete correct Fig. 6 is given below.

**Fig. 6:**
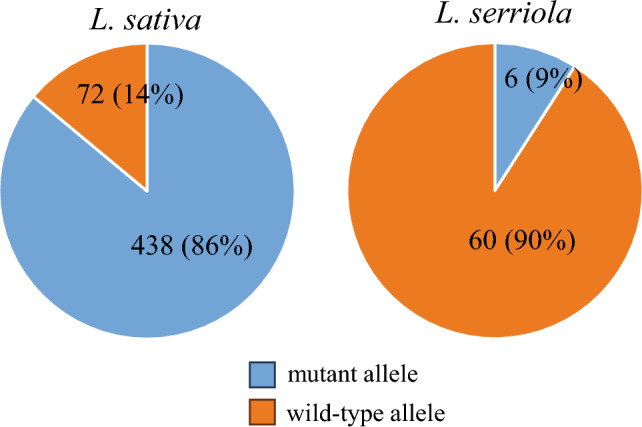
.

The original article has been corrected.

