# Supplementary material for: Low frequency of the wild-type freezing-tolerance LsCBF7 allele among lettuce population suggests a negative selection during domestication and breeding
Source: Theor Appl Genet. 2024 May 18;137(6):135. doi: 10.1007/s00122-024-04643-8 (PMC11420307; doi:10.1007/s00122-024-04643-8)
Supplement: Supplementary file 1 — (DOCX 327 kb) [file 122_2024_4643_MOESM1_ESM.docx]

**Low frequency of the wild-type freezing-tolerance *LsCBF7* allele among lettuce population suggest a negative selection during domestication and breeding**

**Sunchung Park^1,*^, Ainong Shi^2^, and Beiquan Mou^3^**

^1^U.S. Department of Agriculture, Agricultural Research Service, Beltsville, MD 20705, USA

^2^Horticulture Dept., University of Arkansas, Fayetteville, AR 72701, USA

^3^U.S. Department of Agriculture, Agricultural Research Service, Salinas, CA 93905, USA

*Correspondence to Sunchung Park ([Sunchung.park@usda.gov](mailto:Sunchung.park@usda.gov)); ORCID: 0000-0002-7398-9476

Ainong Shi ([ashi@uark.edu](mailto:ashi@uark.edu)); ORCID: 0000-0002-1066-7920

Beiquan Mou ([Beiquan.mou@usda.gov](mailto:Beiquan.mou@usda.gov)); ORCID: 0000-0002-6135-9944

**Supplementary information**

Supplementary Figure S1-4

Supplementary Table S1-6

**
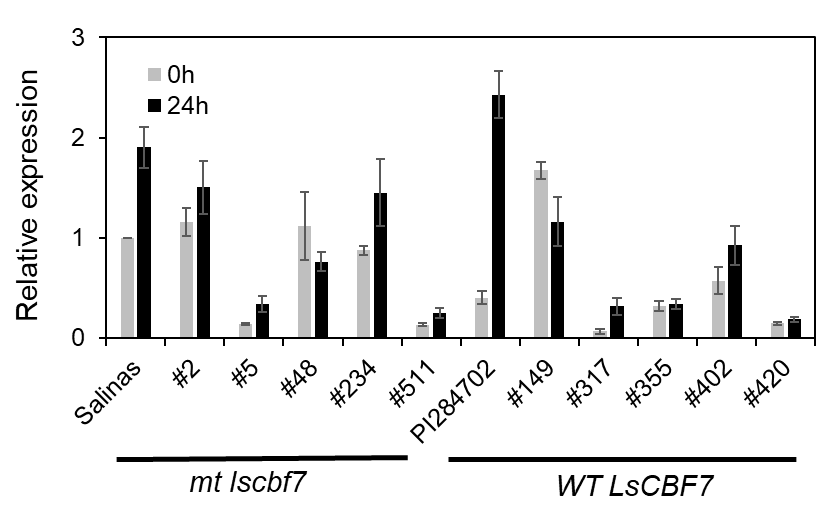
**

**Fig. S1 *LsGOLS2* expression in response to low temperatures in accessions carrying mutant or wild-type LsCBF7 alleles.** Five accessions, each for mutant (*mt lscbf7*) or wild-type (*WT LsCBF7*) accessions, were randomly selected to assess *LsGOLS2* expression. Accession ID numbers are shown on the x-axis. Salinas and PI 284702 were included as controls. Gene expression was determined by qRT-PCR in plants grown at 20°C (0 h) and exposed to 4°C for 24 hours. Error bars represent the standard error of three biological replicates.

**
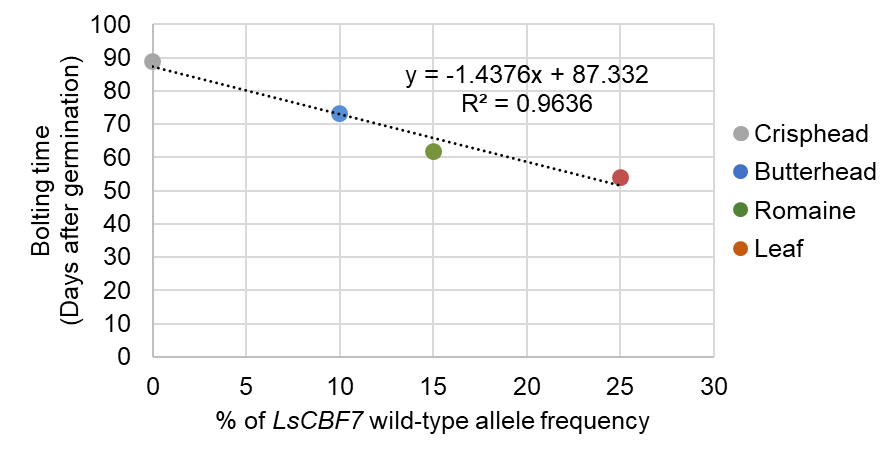
**

**Fig. S2** **Strong negative correlation between the frequency of *LsCBF7* wild-type allele and bolting time in four horticultural types.** The linear regression line and the R-square value are shown on the graph.


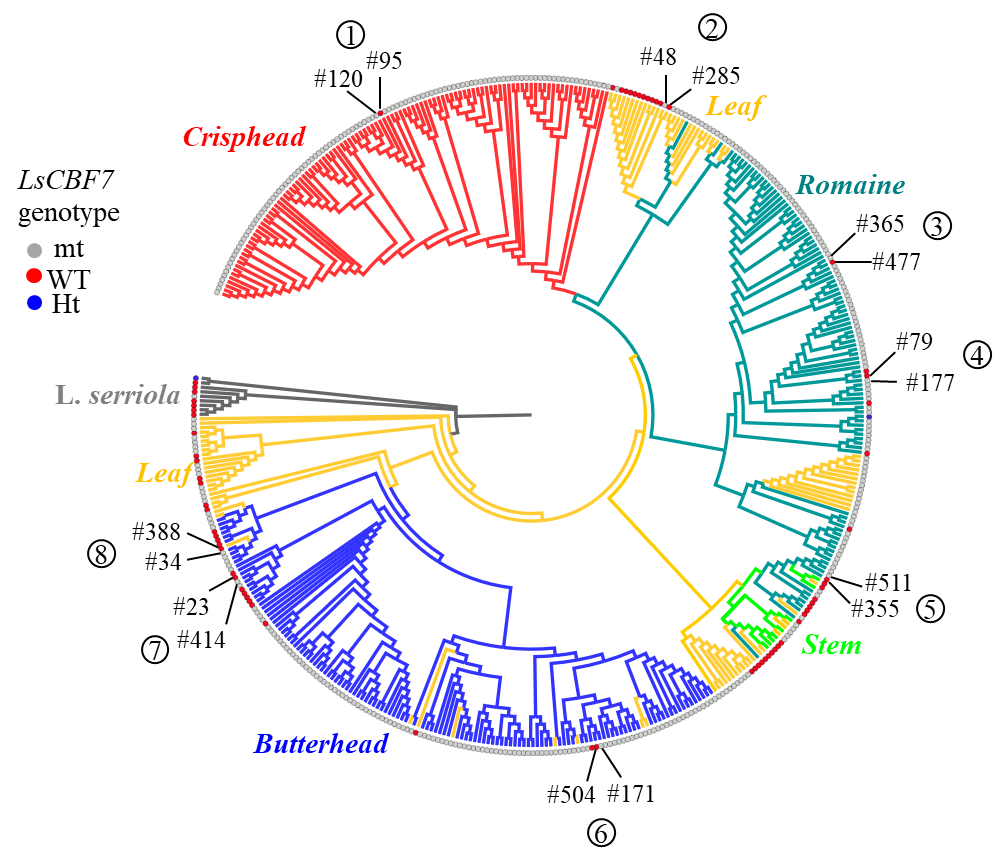


**Fig. S3** **Genetic relationships of 441 accessions and their *LsCBF7* genotypes**. A neighbor-joining tree was previously constructed based on 186,000 SNP (Park et al, 2021), and the *LsCBF7* genotypes (mt-mutant; WT-wild-type; Ht-Heterozygous) identified in this study were overlaid onto each accession. The numbers in the circles represent group identifiers (as shown in Fig. S4) for pairs of accessions, with their corresponding IDs listed below.


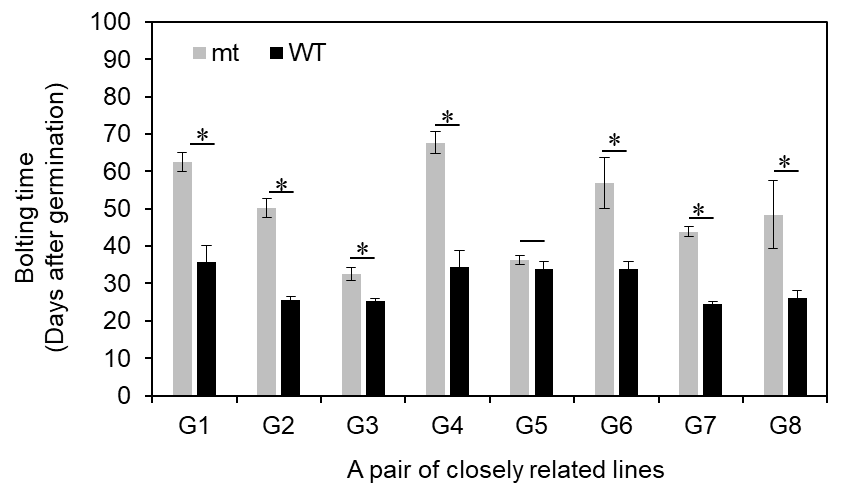


**Fig. S4 Accessions with the wild-type *LsCBF7* allele bolted significantly earlier than those with the mutant allele.** For each pair of genetically closely related accessions, selection was based on the neighbor-joining tree shown in Fig. S3. Error bars indicate standard deviation (n=5). Significance was tested by Student’s t-test (** <0.01, * <0.05).

**Supplementary Tables**

**Table S1.** List of plant species from Asterid or Rosid clades whose protein databases were used for identification of lettuce *GOLS* gene family

**Table S2**. List of genes selected as GOLS-like genes through BLASTP search using Arabidopsis genes as queries

**Table S3.** List of the 577 *Lactuca* accessions used for LsCBF7 allele genotyping

**Table S4.** Primers used for quantitative RT-PCR and rhAmp SNP genotyping

**Table S5.** Genes significantly induced in PI 284702 compared to Salinas plants with log2 fold-change (logFC) >1 and FDR = 0.01

**Table S6.** Pairwise F_ST_ value among *L. serriola* and two *LsCBF7* allelic groups of *L. sativa*

**Table S7.** Heterozygosity (H_exp_) and Shared allele frequency (SAF) among *L. serriola* and two *LsCBF7* allelic groups of *L. sativa*
